# The Conserved Proline18 in the Polerovirus P3a Is Important for Brassica Yellows Virus Systemic Infection

**DOI:** 10.3389/fmicb.2018.00613

**Published:** 2018-04-04

**Authors:** Xiao-Yan Zhang, Tian-Yu Zhao, Yuan-Yuan Li, Hai-Ying Xiang, Shu-Wei Dong, Zong-Ying Zhang, Ying Wang, Da-Wei Li, Jia-Lin Yu, Cheng-Gui Han

**Affiliations:** State Key Laboratory for Agrobiotechnology–Ministry of Agriculture Key Laboratory of Pest Monitoring and Green Management, China Agricultural University, Beijing, China

**Keywords:** brassica yellows virus, P3a, systemic infection, complementation analysis, self-interaction

## Abstract

ORF3a, a newly identified non-AUG-initiated ORF encoded by members of genera *Polerovirus* and *Luteovirus*, is required for long-distance movement in plants. However, the mechanism of action of P3a in viral systemic movement is still not clear. In this study, sequencing of a brassica yellows virus (BrYV) mutant defective in systemic infection revealed two-nucleotide variation at positions 3406 and 3467 in the genome. Subsequent nucleotide substitution analysis proved that only the non-synonymous substitution (C→U) at position 3406, resulting in P3a^P18L^, abolished the systemic infection of BrYV. Preliminary investigation showed that wild type BrYV was able to load into the petiole of the agroinfiltrated *Nicotiana benthamiana* leaves, whereas the mutant displayed very low efficiency. Further experiments revealed that the P3a and its mutant P3a^P18L^ localized to the Golgi apparatus and near plasmodesmata, as well as the endoplasmic reticulum. Both P3a and P3a^P18L^ were able to self-interact *in vivo*, however, the mutant P3a^P18L^ seemed to form more stable dimer than wild type. More interestingly, we confirmed firstly that the ectopic expression of P3a of other poleroviruses and luteoviruses, as well as co-infection with *Pea enation mosaic virus 2* (PEMV 2), restored the ability of systemic movement of BrYV P3a defective mutant, indicating that the P3a is functionally conserved in poleroviruses and luteoviruses and is redundant when BrYV co-infects with PEMV 2. These observations provide a novel insight into the conserved function of P3a and its underlying mechanism in the systemic infection.

## Introduction

Plant viruses are obligate intracellular parasites living exclusively in the symplast of their plant host. In order to establish effectively systemic infection, the viruses that transport in the phloem should fulfill four processes: (1) amplification in the preliminary infection site; (2) short-distance movement between mesophyll cells through the plasmodesmata (PD) (Schoelz et al., 2011); (3) loading into sieve elements after passing through several vasculature-associated cell types and long-distance transport along the vascular bundle system (Carrington et al., 1996; Hipper et al., 2013); (4) virus unloading from sieve elements into distal sink tissues and reestablishment new infection site. In the process of cell-to-cell movement, the viruses (in the form of ribonucleoprotein complex or virions) should successfully open the successive gates of several different cell types including mesophyll cells, bundle sheath, vascular parenchyma cells, companion cells, and then load into sieve elements (Hipper et al., 2013). The viruses are transported along the direction of source-to-sink transportation of carbohydrates in the phloem vasculature and then they exit from sieve elements to establish new infection sites and to disseminate efficiently throughout the whole plant. Two viral forms of transport have been described: virus particles which have capsid protein to protect the genome and RNP complexes composed of viral genome, viral protein, and/or cellular proteins.

Brassica yellows virus (BrYV) is a tentative species in the genus *Polerovirus*. Viruses in this genus and *Luteovirus* are limited to the phloem and transmitted by aphid (King et al., 2012; Zhou et al., 2017). BrYV is distributed widely in mainland China, as well as in South Korea and Japan (Xiang et al., 2011; Zhang et al., 2014; Lim et al., 2015; Kamitani et al., 2016). BrYV can infect and cause leaf malformation and yellowing on cruciferous crops (Xiang et al., 2011; Wang et al., 2015). The BrYVs were divided into three genotypes (BrYV -A, -B, and -C) according to sequence comparisons and phylogenetic analysis (Xiang et al., 2011; Zhang et al., 2014). For convenience of the virus research, the full-length infectious cDNA clones of BrYVs were constructed under control of the cauliflower mosaic virus 35S promoter (Zhang et al., 2015). As the BrYV is a phloem-restricted virus, an umbravirus *Pea enation mosaic virus 2* (PEMV 2) can help BrYV to be mechanically transmitted and invade non-vascular tissue in *Nicotiana benthamiana* (Zhou et al., 2017).

ORF3a was predicted as a newly identified non-AUG-initiated ORF in poleroviruses and luteoviruses by statistical analysis (Smirnova et al., 2015). The amino acid sequence of P3a is generally conserved between polerovirus and luteovirus species. According to the research of *Turnip yellows virus* (TuYV), mutations of the P3a start codon that prevent or increase the expression of P3a (TuYV-3aAGC or TuYV-3aAUG), did not affect TuYV replication and capsid formation, but prevented virus systemic infection (long-distance movement) in plants. The long-distance movement function of TuYV-3aAGC could be restored *in trans* by transiently expressed ORF3a or co-infection of the TuYV-3aAUG. Although P3a was proved to be required for long-distance movement of TuYV, the underlying mechanism is still not clear (Smirnova et al., 2015).

In this study, we identified an artificial BrYV mutant (BrYV^P18L^) which harbored a single amino acid substitution in the P3a (Proline→Leucine at position 18). This mutant loses the ability of systemic infection and cannot load into the petiole of the agro-infiltrated *N. benthamiana* leaves. We found that BrYV-P3a and its mutant localize not only to the Golgi, and near plasmodesmata but also to the endoplasmic reticulum. We also revealed that both BrYV-P3a and its mutant are able to self-interact *in vivo*, but the function deficient mutant P3a^P18L^ seems to form more dimer and to have stronger self-interaction than wild type. We further confirmed that the systemic infection of BrYV P3a mutant can be rescued by ectopically expressed various P3a proteins of other poleroviruses and luteoviruses or by co-infection with PEMV 2.

## Materials and Methods

### Construction of BrYV Full-Length Infectious cDNA and P3a Mutants

The plasmid pTBrA001-3430 was digested with *Stu*I and *Afl*II to obtain a 3328 bp fragment. The plasmid pTBrA3251-*Bgl*3R was digested with *Afl*II and *Bgl*II and the 2338 bp fragment of interest was obtained. Then, these two fragments were ligated between the *Stu*I and *Bam*HI sites of pCB301 to produce the BrYV-A full-length cDNA clone referred to as pCB-BrA. The pCB301 is a binary vector with 2× 35S promoter, ribozyme and NOS terminator (Shen et al., 2014; Zhang P.P. et al., 2017). The mutations were introduced via the inverse-PCR amplification of pTBrA3251-*Bgl*3R using mutagenic primers for PCR (**Supplementary Table S1**). All constructs were verified by sequencing.

### Agroinfiltration of Plants

*Agrobacterium tumefaciens* strain C58CI containing empty pCB301 vector, pCaPEMV 2 (Zhou et al., 2017), pCB-BrA, pCB-BrA derived mutant vectors or protein-expressing pGD vectors were grown at 28°C for 16 h, and infiltrated into 4- to 5-week-old *N. benthamiana* plants. The concentration of the cell suspension was measured by spectrophotometry and adjusted to OD_600_ ≈ 0.5, when mixed infiltrations, OD_600_ was 0.5 for each culture. Upper leaves (0.1 g) were collected 14 dpi for RNA or protein analysis. For analysis of the infiltrated leaves, the samples were collected at indicated time points. Photographs were taken with a Canon (EOS 550D) digital camera.

### Reverse Transcription PCR and Northern Blot Detection

Plant total RNA was prepared by SDS-phenol/chloroform extraction. The 0.1 g leaf samples were ground to fine powder in liquid nitrogen. Before the powdered tissue thawed, 600 μl of phenol:chloroform and 630 μl of extraction buffer (20 mM Tris-HCl, pH 7.8, 1% sodium dodecyl sulfate, 200 mM sodium chloride, and 5 mM EDTA) were added with continuous homogenizing. The thawed mixture was extracted two times with phenol:chloroform and separated by centrifugation. RNA in the supernatant was precipitated by equal volume of 4 M lithium chloride. Then it was washed two times with chilled 75% ethanol and one time with chilled 100% ethanol. The RNA was dissolved in a final volume of 40 μl of diethyl pyrocarbonate-treated water and stored at -20°C for the following protocols.

The reverse transcription (RT) reaction was performed in a 15 μl PCR master mixture consisting of 7.5 μl RNase free ddH_2_O, 3 μl M-MLV RT 5× Buffer (Promega), 1 μl PocoCPR (10 μM), 1 μl dNTP Mixture (each 2.5 mM; TaKaRa), 0.25 μl M-MLV Reverse Transcriptase (200 U/μl; Promega), 0.25 μl Recombinant RNase Inhibitor (40 U/μl; TaKaRa) and 2 μl plant total RNA (∼2 μg). The RT reaction was incubated for 90 min at 37°C (Xiang et al., 2011; Liu et al., 2012). PCR was carried out in a 25 μl mixture containing 12.5 μl 2× TSINGKE Master Mix (blue) (Tsingke Biological Technology Company), 2 μl cDNA template, 0.5 μl of PoconF/PocoCPR primers (10 μM), and 9.5 μl ddH_2_O. Amplified products (10 μl each) were electrophoresed in 1.0% agarose gels and stained with ethidium bromide to confirm the expected size of the fragments.

Five micrograms of RNA were heated in 65°C for 10 min for denaturation and fractionated on a 1.2% formaldehyde-agarose gel and transferred to nitrocellulose (Amersham Hybond-N^+^, GE Healthcare). Prehybridization was performed at 65°C for 5 h in prehybridization buffer (1% BSA, 1 mM EDTA, 7% SDS, 0.5 M Na_2_HPO_4_). The radioactive probe was generated using the Prime-a-Gene labeling system (Promega) and [α – ^32^P] dCTP-labeled DNA probes specific for nt 5161–5620 of BrYV was used for hybridization. After hybridization and washing, the membrane was exposed onto a Phosphoimager screen.

### Western Blot Analysis

Total protein extraction and western blotting were performed as described (Zhuo et al., 2014). Briefly, plant total proteins were extracted by grinding 0.1 g *N. benthamiana* leaves in 300 μl of 2× SDS-PAGE buffer with subsequent heating at 100°C for 10 min. Protein samples were separated by electrophoresis in 12.5% SDS-PAGE and transferred onto Nitrocellulose Membrane (GE Healthcare). Membranes were blocked in 1× TBST buffer (20 mM Tris-HCl pH 7.5, 150 mM NaCl, 0.05% Tween-20) with 5% non-fat milk at 37°C for 1 h, washed three times (10 min each time) with 1× TBST buffer, followed by AP-coupled goat anti-rabbit IgG (Sigma) and NBT (0.33 mg/ml)/BCIP (0.165 mg/ml) staining. BrYV CP, MP, CP-RTD, and PEMV 2 MP were expressed and purified from *Escherichia coli*, and the purified proteins were used for generation of the polyclonal antibodies in rabbits at Institute of Genetics and Developmental Biology, Chinese Academy of Sciences. The antisera raised against BrYV CP, MP, CP-RTD, and PEMV 2 MP were used to detect the accumulation of BrYV and PEMV 2 in *N. benthamiana.* Antisera raised against Flag (Sigma), MBP (GenScript), RFP (GenScript), and GFP (GenScript) were used to detect the expression of P3a fusion proteins.

### Prokaryotic Expression

The P3a and P3a^P18L^ genes were cloned into the prokaryotic expression vector, pDB.His.MBP, to build the pDB.His.MBP-P3a and pDB.His.MBP-P3a^P18L^ fusion protein plasmids. Then, the recombinant plasmids were transformed into *E. coli* Rosetta (DE3) and induced with 0.1 mM isopropyl-β-D-thiogalactopyranoside (IPTG). The expression of the two fusion proteins were analyzed by 12.5% SDS-PAGE and western blotting, respectively. pDB.His.MBP vector was obtained from DNASU Plasmid Repository^1^. The MBP monoclonal antibody in mouse (GenScript) was used to detect the expression of fusion proteins.

### Bimolecular Fluorescence Complementation Assay

To generate BiFC clones, we used the binary vectors pUCSPYNE and pUCSPYCE, which were tagged with the yellow fluorescence protein at the N-terminal region (nYFP) or the C-terminal region (cYFP) (Walter et al., 2004). The ORF3a sequence was inserted into these vectors by the *Xba*I and *Bam*HI enzyme sites, and then the expression vectors were transformed into *A. tumefaciens* strain C58CI. Each expression clone was cultured in LB medium containing 50 mg/L rifampicin and 100 mg/L kanamycin for 12 h and was then harvested by centrifugation. Harvested cells were resuspended in buffer (10 mM MgCl_2_, 10 mM MES, and 0.15 mM acetosyringone) to 0.5 at OD_600_. Resuspended cells were incubated at room temperature for 4 h, and a 2 ml syringe without a needle was used to co-agroinfiltrate *N. benthamiana* leaves with the nYFP expression clone, the cYFP expression clone, and pTBSV-p19. The *N. benthamiana* plants were grown in a growth chamber with a 16/8 day and night photoperiod. The YFP signal was observed by fluorescent microscopy (Olympus, Tokyo, Japan).

### Confocal Laser Scanning Microscopy and Co-localization Assays

We used the binary vectors pGD derivatives pGDGm and pGDRm, which were tagged with the green/red fluorescence protein at the C-terminal region (Goodin et al., 2002; Zhang K. et al., 2017). The ORF3a sequence was inserted into these vectors by the *Xho*I and *Apa*I enzyme sites (**Supplementary Table S1**), and then the expression vectors were transformed into *A. tumefaciens* strain EHA105. Co-expression was performed by agroinfiltration using a bacterial OD_600_ of 0.3 for each. The agrobacteria containing the TBSV-p19 was used at OD_600_ of 0.1. For the visualization of endoplasmic reticulum localization, P3a-RFP and P19 constructs were expressed by leaf agroinfiltration of 16c *N. benthamiana*. These transgenic *N. benthamiana* 16c lines are homozygous for mGFP5-ER expressing green fluorescent protein (GFP) targeted to the ER (Ruiz et al., 1998). The infiltrated leaves were assayed for fluorescence at 2 dpi. High-magnification image were taken with the Olympus FV1000 confocal microscope by using a 60X oil immersion objective lens. Excitation wavelengths were as follows: CFP, 405 nm; GFP, 488 nm; RFP, 546 nm.

### Immunoblot Analyses and Immunoprecipitation

Protein extraction and immunoblot analysis were performed as described previously (Rubio et al., 2005; Hu et al., 2015). For immunoprecipitation, total proteins were extracted from 3 g *N. benthamiana* infiltrated leaves by adding 7 ml protein extraction buffer [25 mM Tris-HCl, pH 7.5, 150 mM NaCl, 1 mM EDTA, 10% glycerol, 10 mM DTT, 2% (w/v) polyvinylpolypyrrolidone (PVPP), 1 mM protease inhibitor cocktail (Sigma-Aldrich), 1% Triton X-100]. Total proteins was mixed with 50 μl of anti-Flag M2 affinity gel (50% suspension; Sigma-Aldrich). After 4 h incubation, the resin was washed three times with IP buffer [25 mM Tris-HCl, pH 7.5, 150 mM NaCl, 1 mM EDTA, 10% glycerol, 1% Triton X-100]. Rabbit polyclonal antibody to the Flag peptide tag (clone M2) and rabbit polyclonal GFP antibody were obtained from GenScript.

### Split-Ubiquitin Membrane-Based Yeast Two-Hybrid System

The DUALmembrane Kit (Dualsystems Biotech), which takes advantage of a split-ubiquitin system, was used for Y2H assays. The ORF3a mutants were cloned into the pBT3-STE bait vector (containing the C-terminal half of ubiquitin) and pPR3-N prey vector (containing the mutated N-terminal half of ubiquitin) using the *Sfi*I sites (Snider et al., 2010). Co-transform of the bait construct and prey vector into yeast reporter strain NMY51. The transformant strains were selected on minimal synthetically defined medium for yeast (SD medium) deprived of leucine and tryptophan for 4 days at 30°C. The positive clones were cultured and plated in 10-fold serial dilutions and grown on higher stringency medium (SD medium deprived of leucine, tryptophan, histidine, and adenine) plates. The bait plasmids co-transformed with pOst1-NubI were used as positive control and co-transformed with pPR3-N were used as negative control.

## Results

### Proline18 in P3a Is Important for BrYV Systemic Infection

**FIGURE 1:**
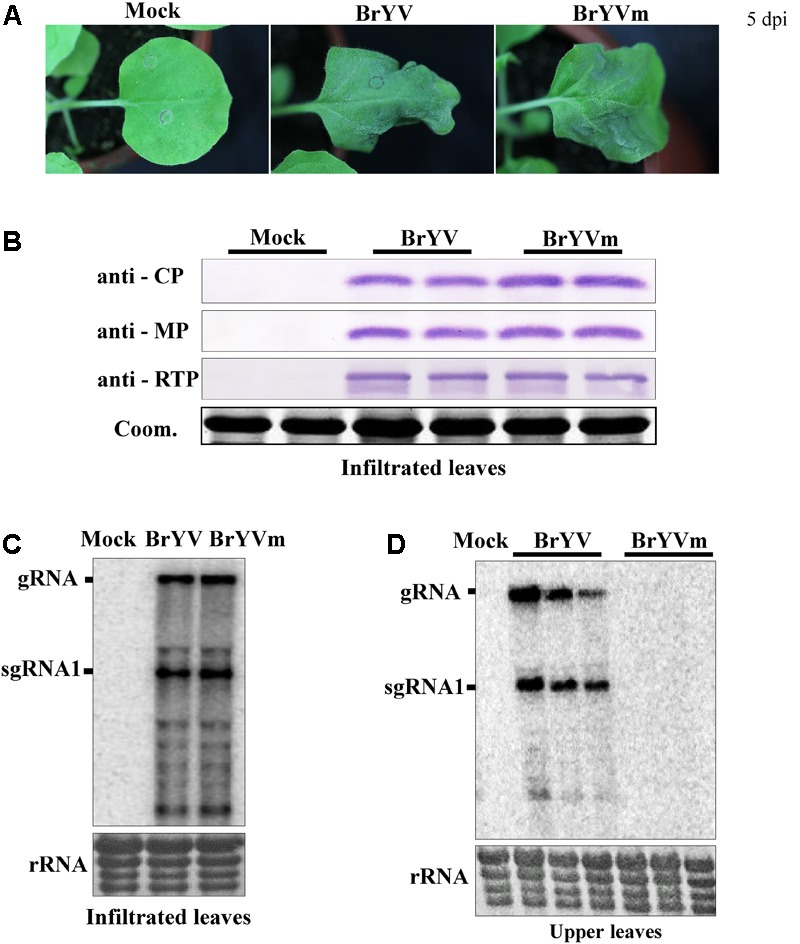
Identification of BrYV systemic infection defective mutant in *Nicotiana benthamiana*. **(A)** Symptoms of BrYV and the mutant (BrYVm) in *N. benthamiana* agroinfiltrated leaves 5 dpi. **(B)** Western blot analysis of the accumulation of BrYV CP, MP, and CP-RTD in BrYV and BrYVm agroinfiltrated *N. benthamiana* leaves at 2 dpi. Coomassie brilliant blue (Coom.) staining is shown as a loading control. Northern blot analysis of RNAs extracted from agroinfiltrated *N. benthamiana* leaves at 2 dpi **(C)** and upper leaves at 14 dpi **(D)**. rRNA, loading control of methylene blue stained ribosomal RNA.

Previously we have developed the infectious cDNA clone of BrYV-A and happened to obtain a BrYV mutant named as “BrYVm.” To examine whether BrYVm is able to move long distance, *N. benthamiana* plants at 3–4 leaf stage were agro-infiltrated with empty vector (Mock), BrYV, and BrYVm, respectively. The agro-infiltrated leaves of BrYVm started to develop necrotic symptoms 5 days post-infiltration (dpi) as BrYV wild type did (**Figure 1A**). At 2 dpi, the inoculated leaves were harvested and extracts subjected to northern blot and western blot analysis. BrYVm genomic and subgenomic RNA species accumulated similarly to wild type (BrYV-WT), and the accumulation of CP, MP, and CP-RTD proteins between BrYV-WT and BrYVm were comparable (**Figures 1B,C**). At 14 dpi, total RNAs extracted from upper leaves were used for northern blot detection (**Figure 1D**). The result showed that BrYV produced gRNA and sgRNA in upper leaves, while BrYVm did not (**Figure 1D**). It can be concluded that BrYVm is not able to move long distance in *N. benthamiana*.

**FIGURE 2:**
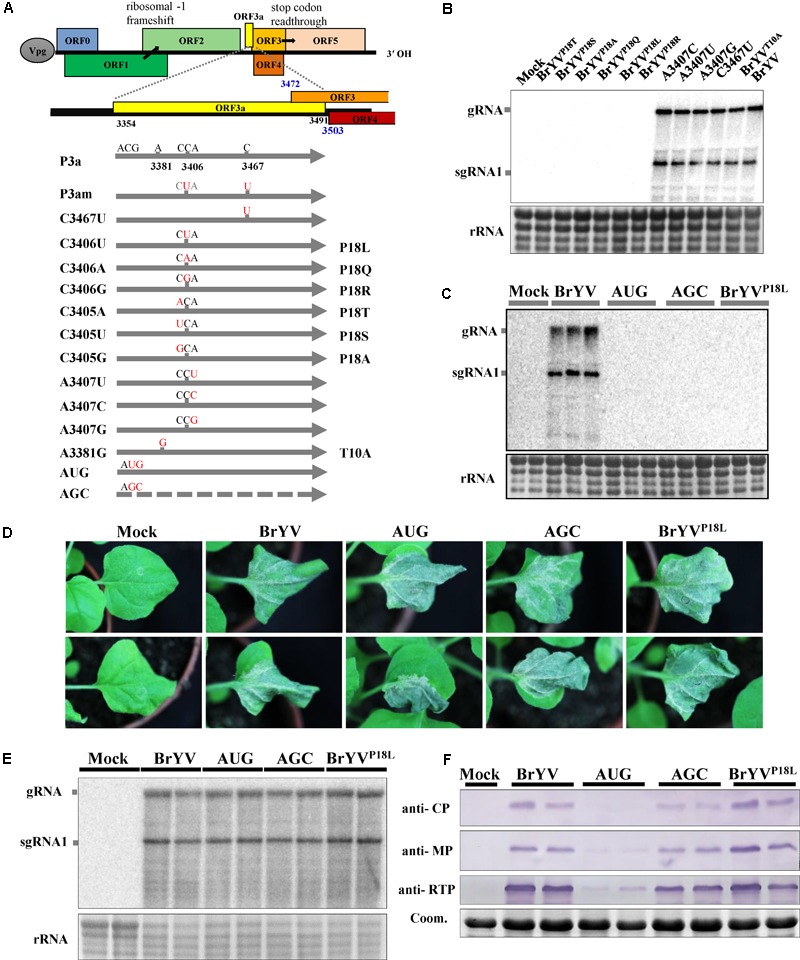
Schematic representation of the BrYV-3a mutants generated and northern blot detection of their systemic infection. **(A)** Representation of BrYV genomic RNA organization. ORFs 0, 1 and 2 are translated from the gRNA with expression of ORF1 being dependent on leaky scanning and translation of ORF2 via a –1 ribosomal frameshift. ORFs 3a, 3, 4, and 5 are translated from sgRNA1, with expression of ORF3a being ACG initiation, of ORF4 via leaky scanning, and of ORF5 via stop codon readthrough. The position of the ORF3a is highlighted in yellow. Nucleotide mutations (red) were introduced into ORF3a and the substitutions are indicated for each mutant (right). **(B,C)** RNAs extracted from *N. benthamiana* upper leaves were submitted to northern blot analyses. The plants infiltrated with the wild-type BrYV or the mutants BrYV^C3405A^ (BrYV^P18T^), BrYV^C3405U^ (BrYV^P18S^), BrYV^C3405G^ (BrYV^P18A^), BrYV^C3406A^ (BrYV^P18Q^), BrYV^C3406U^ (BrYV^P18L^), BrYV^C3406G^ (BrYV^P18R^), BrYV^A3407C^, BrYV^A3407U^, BrYV^A3407G^, BrYV^C3467U^, BrYV^A3381G^ (BrYV^T10A^), BrYV-P3aAUG (AUG), BrYV-P3aAGC (AGC), respectively. B, Each sample corresponds to a mixture of leaves from three individual plants. C. Each sample corresponds to an individual plant. **(D)** Symptoms of *N. benthamiana* infiltrated leaves by agro-infiltration with BrYV mutants observed 5 dpi. **(E)** Northern blot analysis of RNAs extracted from *N. benthamiana* leaves infiltrated with WT or the mutants. **(F)** Western blot analysis of the accumulation of BrYV CP, MP, and CP-RTD in agroinfiltrated *N. benthamiana* leaves at 2 dpi. Coomassie brilliant blue (Coom.) staining is shown as a loading control. Mock, negative control; gRNA, genomic RNA; sgRNA, subgenomic RNA, rRNA, loading control of methylene blue stained ribosomal RNA.

Sequence comparison analysis showed that there are two nucleotides differences between BrYVm and BrYV-WT construct. The C at positions 3406 and 3467 in BrYV were mutated to U, respectively, in BrYVm (**Figure 2A**). Based on the nucleotide sequence of TuYV, we found that translation of BrYV ORF3a started with an ACG (NC_016038.2, nt 3354-3356) and stopped with an UAG (nt 3489-3491), and both of mutation sites mentioned above were located in the internal of ORF3a (**Figure 2A**). In order to define which mutation site is important for the systemic infection of BrYV, two singe nucleotide mutants of BrYV, C3406U, and C3467U, were constructed (**Figure 2A**). Mutant C3406U was made by replacing the C at position 3406 in BrYV with U which lead to leucine substitution of the proline at position 18 of P3a (later named it “BrYV^P18L^”) and the mutant C3467U was generated by changing the C at position 3467 to U which had no effect on the amino acid sequence of P3a (**Figure 2A**). Fourteen days after agro-infiltration of *N. benthamiana* leaves, we found C3467U viral RNA accumulation similar to BrYV-WT in upper leaves while BrYV^P18L^ RNAs were not detected in systemic tissues (**Figure 2B**). In order to investigate whether nucleotide 3406 or its corresponding amino acid Pro18 plays a key role during the systemic infection of BrYV, eight other mutants were constructed (**Figure 2A**). Inoculation and subsequent northern blotting showed that three of the mutants (A3407U, A3407C, and A3407G) which changed the nucleotide without effect on the amino acid sequence of P3a could still infect *N. benthamiana* plants. However, all the Pro18 changing mutants (BrYV^P18Q^, BrYV^P18R^, BrYV^P18T^, BrYV^P18S^, and BrYV^P18A^) lost their capacity of systemic infection as the BrYV^P18L^ (**Figure 2B**). These results strongly suggest that the Pro18 plays an important role in the viral long-distance movement.

To study the function of P3a in the viral long-distance movement, we constructed the P3a-overexpressing BrYV-3aAUG mutant (AUG) and null-3a mutant BrYV-3aAGC (AGC) according to the construction of TuYV-P3a mutants (Smirnova et al., 2015) (**Figure 2A**). By agro-infiltration and northern blotting, we found that neither the AUG nor AGC mutants were able to move long distance at 14 dpi (**Figure 2C**).

To determine whether BrYV^P18L^ plays a role in viral replication, the mutants were inoculated to the *N. benthamiana* plants. At 5 dpi, agro-infiltrated leaves of BrYV^P18L^, AUG and AGC mutants developed similar necrotic symptoms as BrYV did (**Figure 2D**). Northern blotting revealed that BrYV^P18L^, as well as the AUG and AGC, produced gRNA and sgRNA1 at levels similar to that of BrYV-WT (**Figure 2E**). Thus the BrYV^P18L^ has no effect on viral RNA accumulation as well as the AUG and AGC in inoculated leaves of *N. benthamiana* plants. Like the TuYV-3aAUG mutant, BrYV-3aAUG produced drastically decreased accumulation of CP, MP, and CP-RTD proteins. Compared with it, BrYV^P18L^ produced the comparable accumulation of CP, MP, and CP-RTD proteins with those of BrYV-WT and AGC (**Figure 2F**). These results showed that BrYV^P18L^ has no effect on expression of the P3a downstream proteins and the infection in agro-infiltrated *N. benthamiana* leaves.

**FIGURE 3:**
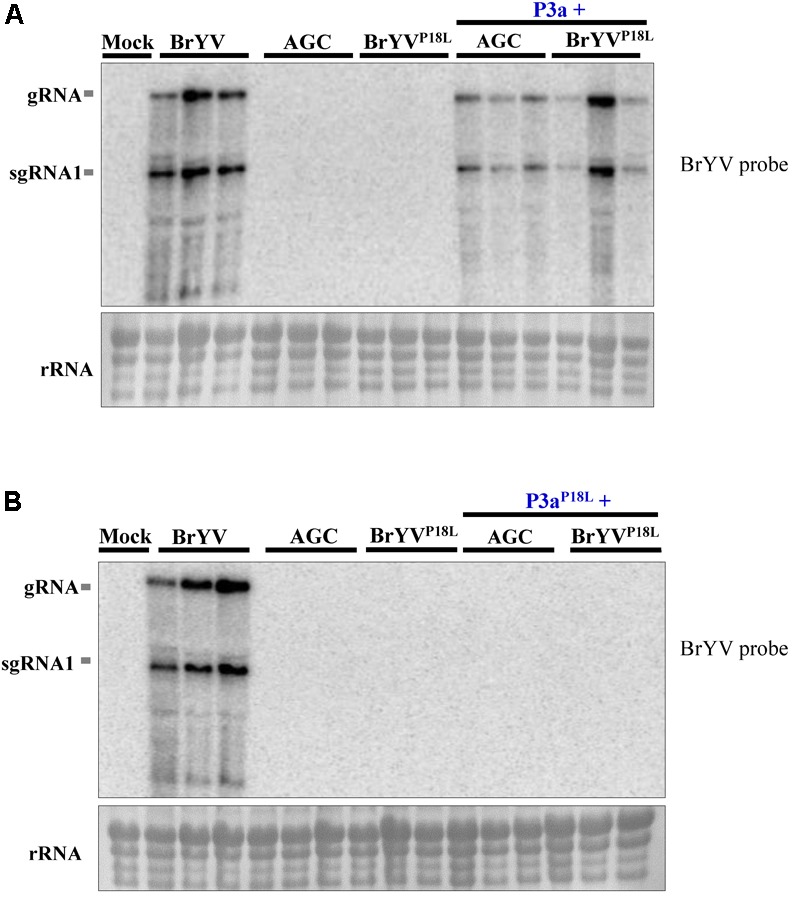
Complementation analysis of BrYV ORF3a mutants. Northern blot detection of RNAs extracted from *N. benthamiana* leaves infiltrated with the BrYV-P3aAGC (AGC), or BrYV^P18L^, and either the agrobacterium transiently expressing the P3a **(A)** or P3a^P18L^ **(B)** protein. Mock, negative control; gRNA, genomic RNA; sgRNA, subgenomic RNA, rRNA, loading control of methylene blue stained ribosomal RNA. Each sample corresponds to an individual plant.

We hypothesize that lack of systemic infection of BrYV^P18L^ is due to the loss of function of P3a in long distance movement. To test the hypothesis, leaves were co-infiltrated with BrYV^P18L^ or AGC and a pGD vector expressing P3a driven by CaMV 35S promoter. Northern blot detection showed that the single infiltration of AGC or BrYV^P18L^ did not lead to systemic infection, while substantial levels of RNA from AGC and BrYV^P18L^ were yielded in systemic leaves of inoculated *N. benthamiana* plants at 14 dpi when co-infiltrated with pGD-P3a (**Figure 3A**). However, no RNA accumulation of AGC or BrYV^P18L^ were detected in upper leaves of inoculated plants when they were co-infiltrated with P3a^P18L^ (**Figure 3B**). Taken together, these results showed that transient expression of P3a can efficiently rescue systemic movement of BrYV^P18L^ or AGC mutants, indicating that the Pro18 residue in P3a is crucial for the long distance movement of BrYV in *N. benthamiana.*

### BrYV^P18L^ Is Not Able to Load Into the Petiole of the Agro-Infiltrated *N. benthamiana* Leaves

**FIGURE 4:**
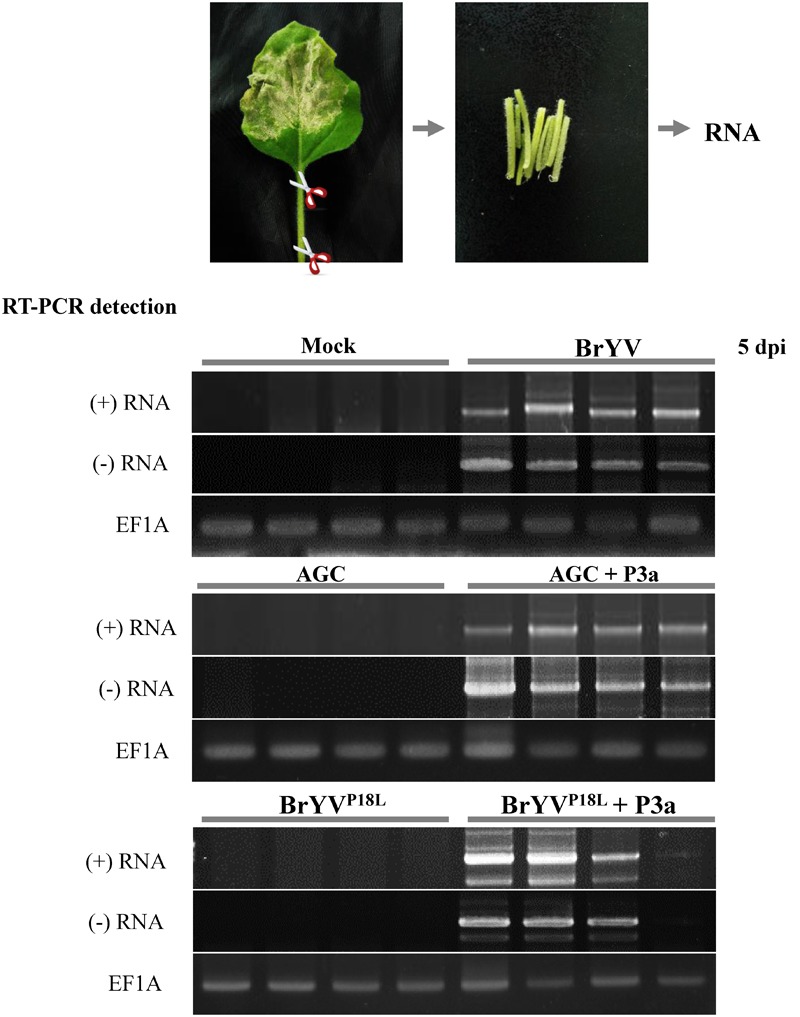
The BrYV P3a mutants BrYV^P18L^ and AGC lost the ability in loading into petiole of the agro-infiltrated *N. benthamiana* leaves. The petioles of the agro-infiltrated *N. benthamiana* leaves with BrYV and its mutants BrYV^P18L^ and AGC were taken at 5 dpi for RT-PCR detection. The RT-PCR detection used the primer PococpR as the reverse transcription primer to detect the (+) RNA and used the primer PoconF as the reverse transcription primer to detect the (–) RNA, and PoconF/PococpR as the PCR amplification primers. Each sample corresponds to two individual plants. The primer 18TR was used as the reverse transcription primer and the primers EF1A-F/EF1A-R were used for PCR detection of the EF1A gene as internal control.

Based on the results mentioned above, we speculated that BrYV^P18L^ and AGC lost the ability to load into phloem vasculature in inoculated tissues, while transiently expressed P3a can help them load into phloem vasculature. To test our hypothesis, we collected the petiole tissues of *N. benthamiana* leaves agro-infiltrated with BrYV^P18L^ or AGC at 5 dpi and conducted RT-PCR detection. The results showed that petiole tissues collected from BrYV-infiltrated leaves were positive for detection of the (+) RNA genome and the (-) RNA genome of BrYV. In contrast, genome RNAs of BrYV^P18L^ or AGC were undetectable in the petiole of infiltrated leaves (**Figure 4**). As shown by the RT-PCR detection results, when co-infiltration with pGD-P3a, both BrYV^P18L^ and AGC mutants could infect petiole tissues of infiltrated *N. benthamiana* leaves at 5 dpi (**Figure 4**).

### P3a and P3a^P18L^ Localize Not Only to Golgi Apparatus, and Near Plasmodesmata, but Also to Endoplasmic Reticulum

**FIGURE 5:**
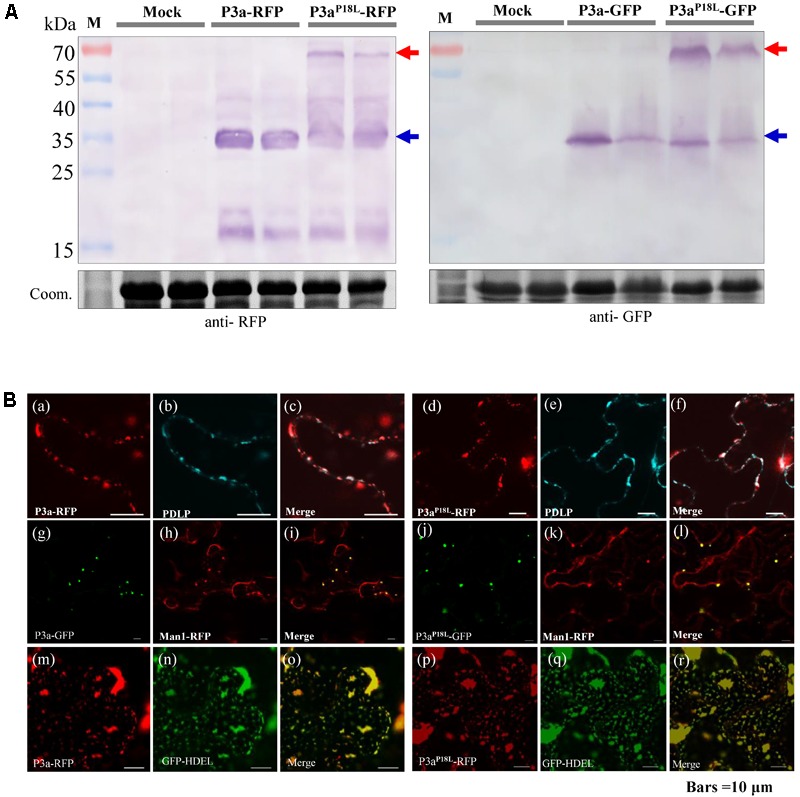
Subcellular localization of P3a in *N. benthamiana* leaves. **(A)** Western blot analysis of P3a-GFP and P3a-RFP in *N. benthamiana* agro-infiltrated leaves. The immunoblots were incubated with antiserum specific to RFP or GFP. Coomassie brilliant blue (Coom.) staining is shown as a loading control. Red and blue arrow marks show that the fusion proteins migrated in their dimeric and monomeric forms, respectively. **(B) a–f**, co-expression of BrYV P3a-RFP or P3a^P18L^-RFP with CFP-PDLP in *N. benthamiana* epidermal tissue. CFP-PDLP was used as a plasmodesmata marker (Li et al., 2017). The CFP (cerulenin fluorescence protein) fluorescence signal is shown as cyan and the RFP fluorescence signal as red. **g–l**, co-expression of BrYV P3a-GFP or P3a^P18L^-GFP with mCherry-based Golgi marker which was used to show Golgi apparatus (Nelson et al., 2007). **m–r**, expression of BrYV P3a-RFP in 16c GFP-HDEL transgenic plants. Experiments were repeated three times and monitored 36 and 48 hpi with similar results. Bars = 10 μm.

In order to explore the possible differences between subcellular localization of P3a and P3a^P18L^, GFP or RFP was fused to the C terminal of P3a. The P3a-GFP/P3a-RFP fusion proteins were transiently expressed in *N. benthamiana* leaves, and their expression was detected by western blotting (**Figure 5A**). Both P3a-GFP and P3a-RFP proteins were visualized by confocal laser scanning microscopy. We confirmed that P3a co-localized with Golgi apparatus and near plasmodesmata shown in the upper and middle panel in **Figure 5B**, as the TuYV-P3a did. The 16c *N. benthamiana* is a transgenic plant which can stably express the known endoplasmic reticulum marker, GFP-HDEL (Ruiz et al., 1998). Interestingly, transiently expressed P3a-RFP in 16c *N. benthamiana* leaves could co-localize with GFP-HDEL (shown at the lower panel in **Figure 5B**), indicating that P3a localized to endoplasmic reticulum as well. Beyond our expectation, the P3a^P18L^ also co-localized with Golgi, endoplasmic reticulum and near plasmodesmata, indicating that the mutant P3a^P18L^ provides no obvious change in subcellular localization of P3a.

### Both P3a and P3a^P18L^ Are Able to Self-interact *in Vivo*, and P3a^P18L^ Accumulates Preferentially as a Dimer Conversely to P3a Wild Type

**FIGURE 6:**
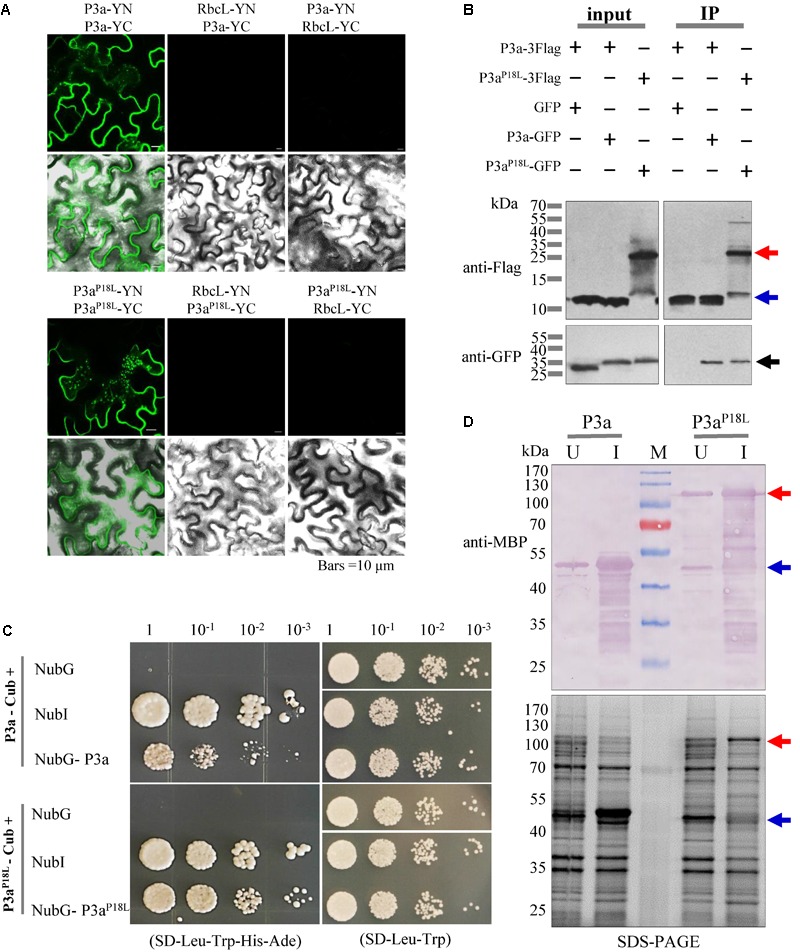
Self-interaction identification of P3a proteins *in vivo*. **(A)** BiFC assays. P3a-YFPN and P3a-YFPC were co-expressed by agro-infiltration in leaf epidermal cells of *N. benthamiana* plants. The upper panel shows a YFP fluorescence image showing the generation of the intracellular fluorescence. The lower panel shows an overlay of a bright-field image and the upper panel. Bars = 10 μm. **(B)** Co-immunoprecipitation immunoblot analysis. Dual combinations of P3a tagged with 3× Flag epitope and GFP epitope were co-infiltrated in *N. benthamiana* leaves and extracts were analyzed at 3 dpi. Co-immunoprecipitation analysis were performed, and the input and immunoprecipitated (IP) proteins were analyzed using anti-Flag and anti-GFP antibodies. The blue arrow indicates monomer and the red arrow indicates dimer. **(C)** The self-interaction of P3a in the split ubiquitin yeast two-hybrid assay. P3a was used as the fused bait protein (P3a-Cub) and the fused prey protein (NubG- P3a). Yeast strain NMY51 co-transformed with the indicated plasmids were subjected to 10-fold serial dilutions, and grown on a SD/-Leu/-Trp/-His/-Ade or SD/-Leu/-Trp medium. The interaction of P3a-Cub/NubI and P3a^P18L^-Cub/NubI are shown as positive control. The interaction of P3a-Cub/NubG and P3a^P18L^-Cub/NubG are shown as negative control. **(D)** Prokaryotic expression of P3a and P3a^P18L^. The prokaryotic expression of His.MBP-P3a and His.MBP-P3a^P18L^ fusion proteins were induced. U, uninduced protein. I, 0.1 mM IPTG induced protein. M, protein marker. The expression of the two fusion proteins were analyzed and identified by 12.5% SDS-PAGE (lower panel) and western blotting (upper panel), respectively. Red and blue arrow marks show that the fusion proteins migrated in their dimeric and monomeric forms, respectively. The MBP monoclonal antibody in mouse was used to detect the expression of fusion protein.

From **Figure 5A**, we noticed that both P3a^P18L^-GFP and P3a^P18L^-RFP showed two bands compared with P3a fusions by western blot detection. According to the molecular weight of the fusion protein, we speculated that the two bands were dimeric and monomeric forms of P3a^P18L^-GFP and P3a^P18L^-RFP. In order to explore whether P3a protein can self-interact *in planta*, bimolecular fluorescence complementation (BiFC) assays and co-immunoprecipitation immunoblot (Co-IP) analysis were carried out. In the BiFC assay, P3a fused to either YN or YC was constructed (P3a-YN, P3a-YC, P3a^P18L^-YN and P3a^P18L^-YC) and then delivered into *N. benthamiana* leaves via agro-infiltration. Pairwise expression of P3a-YN/P3a-YC and P3a^P18L^-YN/P3a^P18L^-YC induced strong YFP signals, which are in the form of numerous tiny fluorescent sites in the cytoplasm of *N. benthamiana* cells at 48 h post infiltration (**Figure 6A**). As expected, no YFP fluorescence was observed when the negative controls P3a-YN/RbcL-YC, RbcL-YN/P3a-YC, P3a^P18L^-YN/RbcL-YC, or RbcL-YN/P3a^P18L^-YC were co-expressed together. BiFC assay revealed that both P3a and P3a^P18L^ have the ability to self-interact *in vivo* (**Figure 6A**).

In Co-IP analysis, P3a-3Flag and P3a-GFP were transiently expressed in *N. benthamiana* leaves via agro-infiltration, and immunoblot analysis were conducted using total proteins from infiltrated leaves. We found that P3a-GFP but not GFP co-precipitated with P3a-3Flag in the presence of anti-Flag beads (**Figure 6B**), indicating a self-interaction of P3a. P3a^P18L^-GFP was also detected in P3a^P18L^-3Flag immunoprecipitate, however, the mutant could be detected in forms of a few monomers and more dimers based on the molecular weight of the fusion proteins under SDS-PAGE denatured separation condition (**Figure 6B**).

Because P3a is a predicted transmembrane protein (Smirnova et al., 2015), the self-interaction of P3a was further investigated by the split-ubiquitin based Membrane Yeast Two-Hybrid (MYTH) system, which is a yeast-based technology to identify protein–protein interactions between integral membrane proteins, membrane-associated proteins and soluble proteins in their natural setting (Snider et al., 2010). As the interaction is detected *in situ* at the membrane, the assay represents a more physiological situation than a conventional yeast two-hybrid assay. According to the DUALmembrane functional assay, co-expression of bait together with Ost1-NubI should result in reconstitution of split-ubiquitin through the strong affinity of wild type Nub for Cub and the concurrent activation of reporter genes. Growth of yeast expressing bait (P3a-Cub) and the NubI control implies that the bait is functional in the DUALmembrane system. Co-expression of bait (P3a-Cub) with the NubG-nonsense peptide fusion expressed from the pPR3-N prey vector did not grow on selective medium. In this system, yeast co-expressing the bait (P3a-Cub) and the prey (NubG-P3a) grew on selective medium indicating that P3a had self-interaction activity (**Figure 6C**). Dilution series allowed the visualization of colony sizes reflecting the efficiency of the interaction and the transcription activation. Therefore, reporter activation appeared more efficient for P3a^P18L^ interaction than P3a-WT (**Figure 6C**).

In addition, the P3a and P3a^P18L^ genes were cloned into the prokaryotic expression vector pDB.His.MBP and the recombinant plasmids were confirmed by DNA sequencing. The results showed that the expression of His.MBP-P3a fusion protein in the form of monomer was detected at 18°C after 20 h of induction with 0.1 mM IPTG. However, the P3a^P18L^ fusion protein was mainly detected in the form of dimer at the same induction condition as confirmed by western blot analysis with the anti-MBP monoclonal antibody (**Figure 6D**).

From these results, we confirmed that both P3a and P3a^P18L^ have the ability to self-interact *in vivo* (**Figure 6**). However, based on the Co-IP, transient expression and prokaryotic expression detection results under SDS-PAGE denatured separation condition, different tagged P3a^P18L^ could be detected in forms of minor monomers and major dimers (**Figures 5A, 6B,D**). Our results strongly suggest P3a^P18L^ forms a more stable dimer than P3a-WT.

### The Systemic Infection of BrYV-3aAGC Can Be Rescued by Ectopically Expressed P3a of Other Poleroviruses and Luteoviruses

**FIGURE 7:**
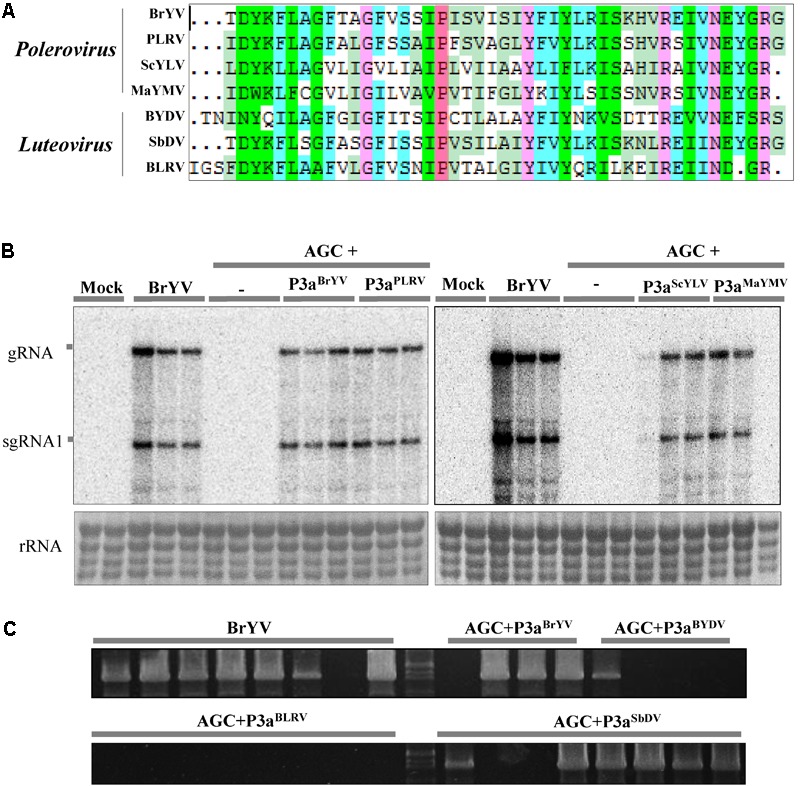
Complementation analysis of P3a from several other poleroviruses and luteoviruses. **(A)** Multiple sequence alignment of seven amino acid sequences of P3a. The conserved proline18 is shown in red. Northern blotting **(B)** and RT-PCR detection **(C)** of systemic infection of the treatments. Co-infiltration of leaves with agrobacteria containing BrYV-3aAGC (AGC) and pGD-P3a^BrYV^, pGD-P3a^PLRV^, pGD-P3a^ScYLV^, pGD-P3a^MaYMV^, pGD-P3a^BYDV-PAV^, pGD-P3a^BLRV^, or pGD-P3a^SbDV^, respectively. Controls were conducted with infiltrations of BrYV-WT, AGC, and the empty vector (Mock). gRNA, genomic RNA; sgRNA, subgenomic RNA, rRNA, loading control of methylene blue stained ribosomal RNA. Each sample corresponds to an individual plant. The RT-PCR detection used the primer PococpR as the reverse transcription primer and PoconF/PococpR as the PCR amplification primers.

Since the amino acid sequences of P3a are conserved among diverse members in *Polerovirus and Luteovirus*, we tested whether other P3a can also complement the systemic movement of AGC. We subcloned the ORF3a coding sequences of *potato leafroll virus* (PLRV, KP090166), *sugarcane yellow leaf virus* (ScYLV), maize yellow mosaic virus (MaYMV), *bean leafroll virus* (BLRV, NC_003369.1), *barley yellow dwarf virus-PAV* (BYDV-PAV), and *soybean dwarf virus* (SbDV, NC_003056.1) into the pGD vector. The amino acid sequences of different P3a proteins are shown in **Figure 7A**, as it can be observed the Pro at position 18 is strictly conserved in all polero- and luteoviruses analyzed. We performed co-infiltration in *N. benthamiana* plants with agrobacteria containing AGC mutant and pGD-P3a^PLRV^, pGD-P3a^ScY LV^, or pGD-P3a^MaY MV^, followed by northern blot detection at 14 dpi. We found that all the inoculated plants yielded substantial levels of viral RNAs in systemic leaves, illustrating that P3a^PLRV^, P3a^ScY LV^, or P3a^MaY MV^ could indeed rescue the systemic movement of AGC (**Figure 7B**). RT-PCR detection results showed that transient expression of P3a^BY DV^ and P3a^SbDV^ could also help AGC mutant to infect the upper leaves (**Figure 7C**). In all the complementation experiments, no base changes in the ORF3a region were observed in the progeny of the mutant. It can be concluded that different P3a of poleroviruses and luteoviruses have a conserved function in viral systemic infection.

### BrYV ^P18L^ and AGC Can Be Complemented by PEMV 2 Co-infection

**FIGURE 8:**
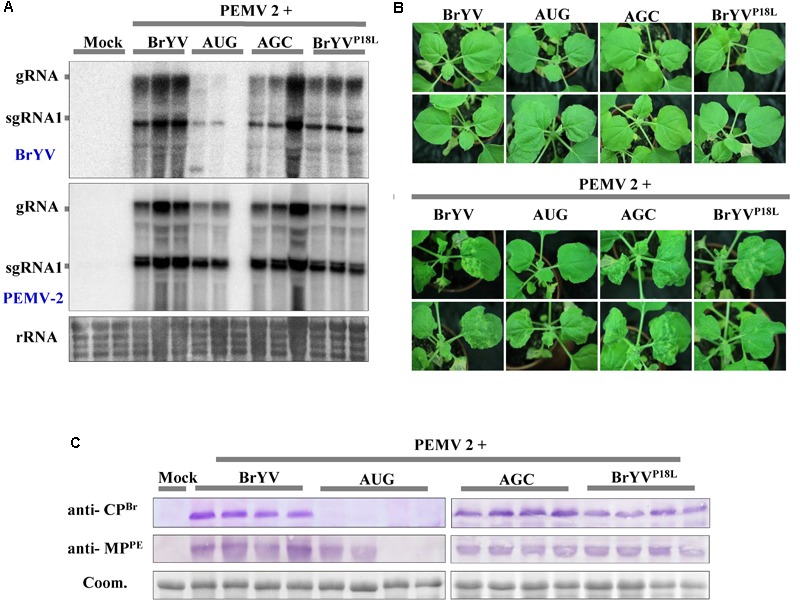
Systemic infection after leaves co-infiltration of BrYV P3a mutants and *Pea enation mosaic virus 2* (PEMV 2). **(A)** Northern blotting of RNAs extracted from different treated *N. benthamiana* upper leaves 14 dpi. rRNA, loading control of methylene blue stained ribosomal RNA. **(B)** Western blotting analyses of the accumulation of BrYV CP and PEMV 2 MP extracted from *N. benthamiana* upper leaves 14 dpi using specific antisera raised against BrYV CP and against PEMV 2 MP. Coomassie brilliant blue (Coom.) staining is shown as a loading control. **(C)** The symptoms of *N. benthamiana* upper leaves by co-infiltration with full-length cDNA clones of PEMV 2 and BrYV or its mutants.

Brassica yellows virus is restricted to the phloem tissue and it can invade non-vascular tissues when co-infected with PEMV 2. Co-infection of BrYV and PEMV 2 produced severe symptoms and increased the accumulation of BrYV in upper leaves of *N. benthamiana* (Zhou et al., 2017). In order to identify whether the PEMV 2 could also effectively complement the long-distance movement of the BrYV mutants (BrYV^P18L^, AUG, and AGC), *N. benthamiana* plants were co-infiltrated with the mutants and PEMV 2, respectively. Northern blot and western blot detection showed that all the mutants successfully infected the upper leaves of *N. benthamiana* in the presence of PEMV 2 (**Figures 8A,B**). At 14 dpi, BrYV^P18L^ + PEMV 2 and AGC + PEMV 2 both showed the severe chlorotic and leaf curling symptoms in the upper leaves of inoculated *N. benthamiana*, which is similar to that of the BrYV + PEMV 2 in inoculated plants (**Figure 8C**). However, upper leaves of plants co-infected with AUG + PEMV 2 showed weaker chlorotic symptom compared with BrYV + PEMV 2 infected plants (**Figure 8C**), consistent with the results of molecular detection. In this complementation assay, sequence progeny for mutants (BrYV^P18L^, AUG, and AGC) accumulating in upper leaves was determined by RT-PCR followed by amplicon sequencing. All the mutations were retrieved indicating the absence of revertants or pseudorevertants in the P3a sequence. In addition, the mutants (BrYV^P18R^ or BrYV^P18Q^) also regained the ability of long distance movement and exhibited severe symptoms in the presence of PEMV 2 (**Supplementary Figure S1**). These experiments showed that PEMV 2 could complement function of BrYV P3a in virus systemic infection.

## Discussion

According to the research on TuYV P3a, the P3a is not required for virus replication in *Chenopodium quinoa* protoplasts, but is essential for viral long distance movement (Smirnova et al., 2015). Here, our data demonstrated that BrYV mutants with mutations in P3a (BrYV^P18L^ and AGC) lost efficient systemic infection in the *N. benthamiana* without effect on the viral accumulation in inoculated leaves. The deficiency of BrYV mutants in long distance trafficking can be retrieved with the help of ectopically expressed P3a, indicating an essential role of P3a in BrYV systemic infection. The statement that Pro18 of P3a plays an important role in the long-distance movement of BrYV could be supported by two lines of evidence: (1) Substitution analysis revealed that Pro18 substitution in P3a abolished systemic infection of BrYV in *N. benthamiana*, (2) Transient expression of P3a could efficiently rescue systemic movement of the mutant in complementation assay. More interestingly, Pro18 is conserved in the alignment of 459 luteovirid sequences of ORF3a done by Smirnova et al. (2015) (supplementary data) in all but one sequence. The single exception is the isolate of BrYV with the P18L mutation studied here, indicating that this large alignment provides powerful evidence of the requirement for Pro18. The importance of a Pro residue in a MP in the viral movement has already been described for a plant virus (Martinez-Gil et al., 2009) and for animal viruses (Gómara et al., 2004), in which the substitution of proline has been shown to abolish infectivity and to interfere with the ability to induce bilayer destabilization. It is well known that the presence of a proline in the hydrophobic region constrains the transmembrane (TM) disposition, the secondary structure, and its interaction with lipid interfaces (Genovés et al., 2011) making these apparent TM domains not really true but only associated to membrane. It is possible that the Pro18 serves as a key residue of the P3a protein motif or structure, and substitution of Pro18 with Leu may result in a conformational change that renders P3a protein non-functional. We speculate that P3a functions in the cell where it is expressed and helps the virus to load into the phloem tissues. BrYV^P18L^ or AGC could not enter the petiole of the agro-infiltrated *N. benthamiana* leaves, but they were easily detected from petiole tissues of the agro-infiltrated *N. benthamiana* when co-infiltrated with pGD-P3a. Our findings may provide important new implications to guide future mechanistic studies on how the P3a protein helps BrYV load into phloem vasculature and leads to systemic infection.

Both P3a and P3a^P18L^ have the self-interaction activity, however, whether the self-interaction of P3a is necessary for BrYV systemic infection is uncertain. Compared with the wild type, the mutant P3a^P18L^ seems to form dimers so stable that they are not separated even under denaturing gel electrophoresis conditions used for the western blots. This implies that P3a^P18L^ may interfere with the dissociation step of a dynamic process of dimer formation and dissociation (Durand et al., 2012; Zoued et al., 2018). Although leucine substitution of Pro18 is unable to affect the subcellular localization of P3a, the multimerization of P3a^P18L^ may affect other biological functions of P3a, for instance its interaction with other viral or host factors. As noted already, the plant ER is interconnected among the cells via the desmotubule of the PD, forming a continuous ER network throughout the plant (Stefano et al., 2014). The ER membrane also serves as an important platform for anchoring several other viral MPs, which are required for intercellular movement and viral spread (Krishnamurthy et al., 2002; Ju et al., 2005; Kaido et al., 2009, 2014; Wu et al., 2011; Feng et al., 2016). P3a localized on both the ER-Golgi and PD, the virus likely recruits essential host factors or viral proteins to facilitate its loading into phloem tissue. As CP, MP, and CP-RTD participate in the movement of poleroviruses (Lee et al., 2002, 2005; Kaplan et al., 2007; Peter et al., 2008; Hipper et al., 2014), P3a may act in concert with CP, MP, CP-RTD, or host proteins to help the virus load into phloem tissue and move long distance in the phloem.

The amino acid sequences of P3a are conserved among poleroviruses and luteoviruses (Smirnova et al., 2015). P3a^BrYV^ shared 44.2–69.8% identities with those of P3a^PLRV^, P3a^ScYLV^, P3a^MaYMV^, P3a^BYDV^, or P3a^SbDV^. Our results revealed for the first time that ectopical expression of these proteins are able to help AGC mutant systemically infect *N. benthamiana* plants as P3a^BrYV^. In the complementation assay, P3a^BLRV^ could not complement the movement of AGC mutant. However, we are unable to rule out the possibility that the BLRV ORF3a we cloned is function-deficient, since no BLRV infectious clone has been obtained yet. In conclusion, our results indicated that the P3a of poleroviruses and luteoviruses is evolutionarily and functionally conserved.

Viral synergism occurs commonly in nature. It usually increases the accumulation of one of the doubly infected viruses in the plant by facilitating its replication (Vance, 1991; Karyeija et al., 2000) or movement to more tissues (Barker, 1987; Zhou et al., 2017), or causes more severe symptoms in hosts than those caused by each single infection (Untiveros et al., 2007; Syller, 2012). Synergistic interactions between poleroviruses and umbraviruses have destructive effects on crop plants (Naidu et al., 1998; Mo et al., 2002). In previous studies, we have described that the *N. benthamiana* plants that co-infiltrated with BrYV and PEMV 2 developed mild leaf curling and produced chlorotic spots at 14 dpi. Molecular detection showed that the accumulation of BrYV RNAs and CP in *N. benthamiana* plants was greatly increased compared to that in singly infected plants. Moreover, BrYV can be mechanically transmitted with the help of PEMV 2. *In situ* hybridization assay showed that PEMV 2 can help BrYV invade non-vascular tissues, as the BrYV alone is limited to the vascular tissues (Zhou et al., 2017). Neither BrYV^P18L^ nor AGC is capable of systemic infection individually, but PEMV 2 can complement their systemic infection in *N. benthamiana* plants with equivalent efficiency of BrYV wild type. The data provided in this work strongly support the notion that PEMV 2 not only plays a role as P3a in facilitating long distance movement of AGC, but also interacts with AGC synergistically in the absence of P3a. This fact implies that the process in which PEMV 2 helps BrYV transport from the phloem into the mesophyll cells does not need P3a. Similarly, *Pea enation mosaic virus 1* (PEMV1, *Enamovirus* genus in the *Luteoviridae* family) which does not contain ORF3a and ORF4 (both present in luteoviruses and poleroviruses) relies on PEMV2 to move long distance in the plants (Demler et al., 1993, 1994). Therefore, in the synergistic interaction between BrYV and PEMV 2, the P3a is redundant in long-distance movement.

## Conclusion

We identified the Pro18 as an important residue for function of P3a in viral long distance movement. BrYV P3a defective mutants were not able to load into the petiole of the agroinfiltrated *N. benthamiana* leaves. However, they could establish efficient systemic infection by ectopically expressing P3a, as well as co-infection with PEMV 2. These data indicated that the P3a is functionally conserved in poleroviruses and luteoviruses, and that function of P3a is redundant when BrYV co-infects with PEMV 2. These observations provide a novel insight into the function of P3a. However, there are still many questions about P3a to be answered: how does P3a help BrYV load into phloem tissues? Is the self-interaction of P3a essential for the systemic infection? Is the P3a required for aphid transmission? Identifying the host proteins or viral proteins that interact with P3a will be the focus of our future investigations.

## Author Contributions

C-GH conceived the study and revised the manuscript critically. X-YZ and T-YZ carried out the experiments. X-YZ drafted the manuscript and assembled the pictures. H-YX and S-WD constructed the several plasmids. Y-YL revised the manuscript. Z-YZ, YW, D-WL and J-LY contributed the reagents/materials/analysis tools. All authors have read and approved the final manuscript.

## Conflict of Interest Statement

The authors declare that the research was conducted in the absence of any commercial or financial relationships that could be construed as a potential conflict of interest.

## Footnotes

1 https://dnasu.org/DNASU/Home.do

## Supplementary Material

FIGURE S1 Systemic infection of BrYV P3a mutants can be rescued by PEMV 2 co-infection. **(A)** Western blotting of the accumulation of BrYV CP and PEMV 2 MP extracted from *N. benthamiana* upper leaves at 14 dpi. Agro-infiltration was performed at an OD_600_ of 0.5 (when mixed infiltrations, OD_600_ was 0.5 for each culture). Antisera raised against BrYV CP and against PEMV 2 MP were used for detection. Coomassie brilliant blue (Coom.) staining is shown as a loading control. **(B)** The symptoms of *N. benthamiana* upper leaves by co-infiltration with PEMV 2 and BrYV or its mutants.

TABLE S1 Sequences of primers used in this study.
