# Supplementary figures and images for: The Conserved Proline18 in the Polerovirus P3a Is Important for Brassica Yellows Virus Systemic Infection
Source: Front Microbiol. 2018 Apr 4;9:613. doi: 10.3389/fmicb.2018.00613 (PMC5893644; doi:10.3389/fmicb.2018.00613)

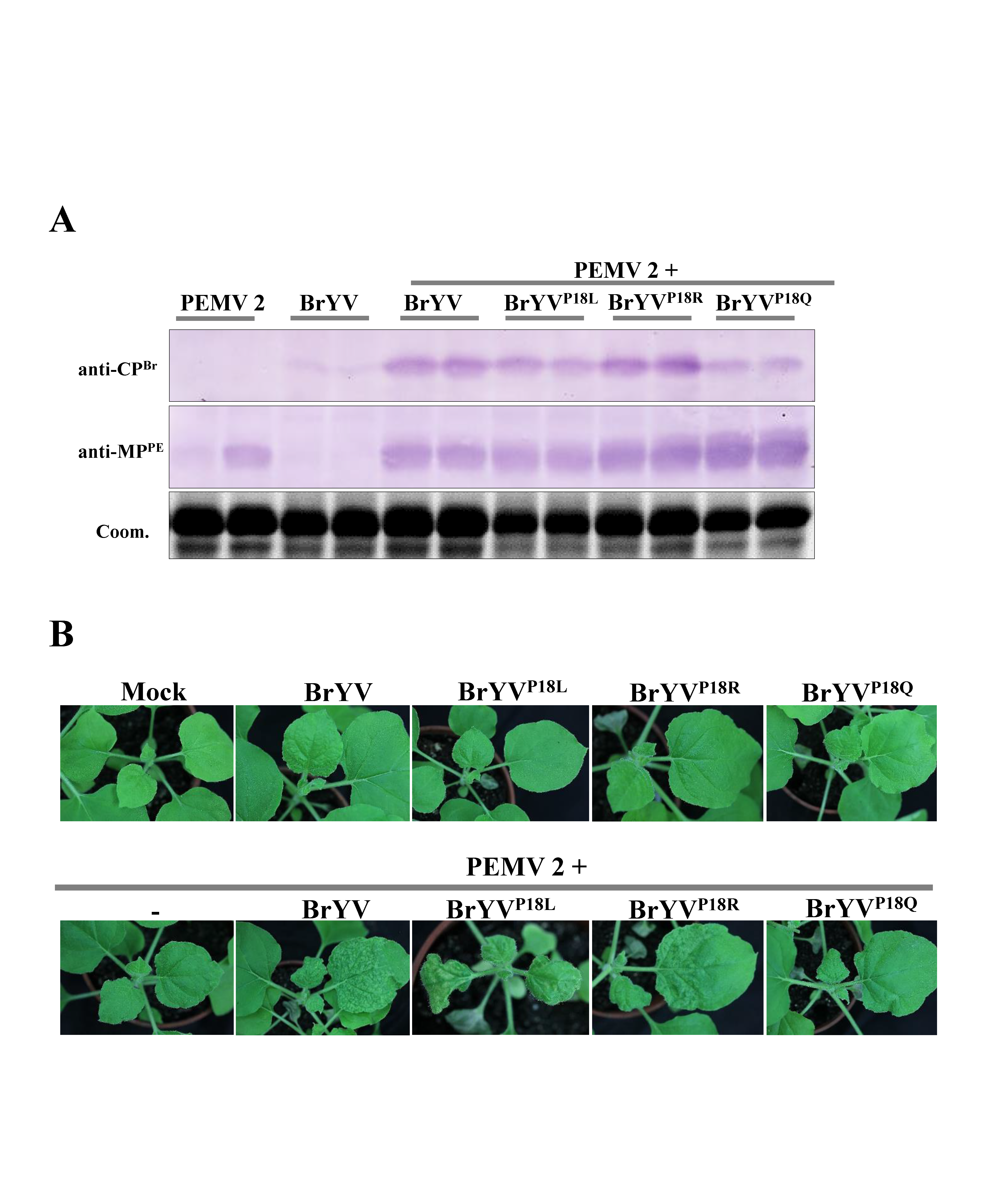

Supplement: FIGURE S1 — Systemic infection of BrYV P3a mutants can be rescued by PEMV 2 co-infection. (A) Western blotting of the accumulation of BrYV CP and PEMV 2 MP extracted from N. benthamiana upper leaves at 14 dpi. Agro-infiltration was performed at an OD600 of 0.5 (when mixed infiltrations, OD600 was 0.5 for each culture). Antisera raised against BrYV CP and against PEMV 2 MP were used for detection. Coomassie brilliant blue (Coom.) staining is shown as a loading control. (B) The symptoms of N. benthamiana upper leaves by co-infiltration with PEMV 2 and BrYV or its mutants. [file Image_1.TIFF]
