# Supplementary material for: The Conserved Proline18 in the Polerovirus P3a Is Important for Brassica Yellows Virus Systemic Infection
Source: Front Microbiol. 2018 Apr 4;9:613. doi: 10.3389/fmicb.2018.00613 (PMC5893644; doi:10.3389/fmicb.2018.00613)
Supplement: TABLE S1 — Sequences of primers used in this study. [file Table_1.DOCX]

**Supplementary Table 1 Sequences of primers used in this study**

| Experiment | Name | Primer sequence ^a^ | Note |
| --- | --- | --- | --- |
| Cloning of BrYV P3a mutants | BrA3aATG/F | ATGGATTACAAATTCCTAGC | pCB301-BrYV-P3aAUG |
|  | BrA3a3253/R | TGTATCTTTGAGTGGTTTTACT |  |
|  | BrA3aAGC/F | AGCGATTACAAATTCCTAGC | pCB301-BrYV-P3aAGC |
|  | BrA3a3253/R | TGTATCTTTGAGTGGTTTTACT |  |
|  | BrAC3467TF | CGAAATTGTTAATGAATACGGTCGTG | pCB301-BrYV^C3467U^ |
|  | BrAC3467TR | CGTACGTGTTTAGAGATTCTTAGGTAG |  |
|  | BrAC3406TF | GATACTAATATCCGTGATCAGTATC | pCB301-BrYV^P18L^ |
|  | BrAC3406TR | GATGAAACGAAGCCTGCGGTGAAAC |  |
|  | BrAC3406AF | CAAATATCCGTGATCAGTATCTATTTC | pCB301-BrYV^P18Q^ |
|  | BrA3a3404R | TATCGATGAAACGAAGCCTGCGGTG |  |
|  | BrAC3406GF | CGAATATCCGTGATCAGTATCTATTTC | pCB301-BrYV^P18R^ |
|  | BrA3a3404R | TATCGATGAAACGAAGCCTGCGGTG |  |
|  | BrAC3405AF | ACAATATCCGTGATCAGTATCTATTTC | pCB301-BrYV^P18T^ |
|  | BrA3a3404R | TATCGATGAAACGAAGCCTGCGGTG |  |
|  | BrAC3405TF | TCAATATCCGTGATCAGTATCTATTTC | pCB301-BrYV^P18S^ |
|  | BrA3a3404R | TATCGATGAAACGAAGCCTGCGGTG |  |
|  | BrAC3405GF | GCAATATCCGTGATCAGTATCTATTTC | pCB301-BrYV^P18A^ |
|  | BrA3a3404R | TATCGATGAAACGAAGCCTGCGGTG |  |
|  | BrAA3407CF | CCCATATCCGTGATCAGTATCTATTTC | pCB301-BrYV^A3407C^ |
|  | BrA3a3404R | TATCGATGAAACGAAGCCTGCGGTG |  |
|  | BrAA3407TF | CCTATATCCGTGATCAGTATCTATTTC | pCB301-BrYV^A3407U^ |
|  | BrA3a3404R | TATCGATGAAACGAAGCCTGCGGTG |  |
|  | BrAA3407GF | CCGATATCCGTGATCAGTATCTATTTC | pCB301-BrYV^A3407G^ |
|  | BrA3a3404R | TATCGATGAAACGAAGCCTGCGGTG |  |
| Cloning of subcellular localization vectors of P3a | BrP3aXhoF | **CTCGAG**ATGGATTACAAATTC | pGDGm-P3a, pGDRm-P3a, pGDGm-P3a^P18L^, pGDRm-P3a^P18L^ |
|  | BrP3aApaR | **GGGCCC**CCCACGACCGTATT |  |
| Cloning of expression vectors of different P3a | BrP3aXhoF | **CTCGAG**ATGGATTACAAATTC | pGD-P3a, pGD-P3a^P18L^, pGD-P3a-3Flag, pGD-P3a^P18L^-3Flag |
|  | BrP3aApa3R | **GGGCCC**CTACCCACGACCGTATT |  |
|  | PLP3aXhoF | **CTCGAG**ATGGATTATAAATTCT | pGD-P3a^PLRV^ |
|  | PLP3aApa3R | **GGGCCC**TTAACCACGACCGTACTC |  |
|  | MaYMV-P3aXhoF | **CTCGAG**ATGGATTGGAAACTCTTTTG | pGD-P3a^MaYMV^ |
|  | MaYMV-P3aApa3R | **GGGCCC**CTACCTCCCGTATTCAT |  |
|  | ScYLV-P3aXhoF | **CTCGAG**ATGGATTACAAACTTCT | pGD-P3a^ScYLV^ |
|  | ScYLV-P3aApa3R | **GGGCCC**TTAGCGCCCGTATTCA |  |
|  | PAV-P3aXhoF | **CTCGAG**ATGAATATTAATTACCAA | pGD-P3a^BYDV-PAV^ |
|  | PAV-P3aApa3R | **GGGCCC**CTACGACCTACTGAAT |  |
|  | BLRV-P3aXhoF | **CTCGAG**ATGGGTTCCTTCGATTA | pGD-P3a^BLRV^ |
|  | BLRV-P3aApaR | **GGGCCC**CTAGCGACCATCGTTGATT |  |
|  | SbDV-P3aXhoF | **CTCGAG**ATGGATTACAAATTCCTA | pGD-P3a^SbDV^ |
|  | SbDV-P3aApaR | **GGGCCC**CTAACCGCGACCATACT |  |
| Cloning of BiFC vectors | BrP3aXbaF | **TCTAGA**ATGGATTACAAATTC | pUCSPYNE-P3a, pUCSPYCE-P3a, pUCSPYNE-P3a^P18L^, pUCSPYCE-P3a^P18L^ |
|  | BrP3aBamHR | **GGATCC**CCCACGACCGTATT |  |
| Cloning of Y2H vectors | BrP3aSfiF | ATTAACAA**GGCCATTACGGCC**GATTACAAATTCCTAGC | pBT3-STE-P3a, pBT3-STE-P3a^P18L^ |
|  | BrP3aSfiR-1 | AACTGATT**GGCCGAGGCGGCC**CCCCCACGACCGTATTCA |  |
|  | BrATGP3aSfiF | ATTAACAA**GGCCATTACGGCC**ATGGATTACAAATTCCT | pPR3-N-P3a, pPR3-N-P3a^P18L^ |
|  | BrP3aSfi3R-1 | CTCGAGA**GGCCGAGGCGGCC**CTACCCACGACCGTATT |  |
| Cloning of prokaryotic expression vectors | 3aNdeF | **CATATG**ACGGATTACAAATTC | pDB.His.MBP-P3a, pDB.His.MBP- P3a^P18L^ |
|  | 3aXho3R | **CTCGAG**CTACCCACGACCGTATT |  |

^a^ The underlined letters represent the site of mutation. The bold font represent the restriction sites.
